# Biodegradation of Heterogeneous Industrial Multi-Walled Carbon Nanotubes by Pro-Inflammatory Macrophages

**DOI:** 10.3390/nano14201616

**Published:** 2024-10-10

**Authors:** Alexander G. Masyutin, Ekaterina K. Tarasova, Daniil A. Samsonov, Galina E. Onishchenko, Maria V. Erokhina

**Affiliations:** 1 Department of Cell Biology and Histology, Faculty of Biology, Lomonosov Moscow State University, 1-12 Leninskie Gory, Moscow 119991, Russia; samsamson1317@gmail.com (D.A.S.); galina22@mail.ru (G.E.O.); masha.erokhina@gmail.com (M.V.E.); 2 Department of Pathology, Cell Biology and Biochemistry, Central Tuberculosis Research Institute, 2 Yauzskaya Alleya, Moscow 107564, Russia; shalioto6@gmail.com

**Keywords:** industrial-grade multi-walled carbon nanotubes, MWCNT, biodegradation, proinflammatory macrophages

## Abstract

Industrial multi-walled carbon nanotubes (ig-MWCNTs) make up the majority of carbon nanomaterials, and human contact with them is the most probable. At the same time, the biodegradation of ig-MWCNTs by phagocytes has not been studied—existing articles consider mainly laboratory-grade/functionalized MWCNTs (l-MWCNTs), in contrast to which ig-MWCNTs are a highly heterogeneous nanomaterial in terms of morphological and physicochemical characteristics. The aim of the present study was to analyze ig-MWCNTs’ biodegradation by proinflammatory macrophages. We focused on both extra- and intracellular ig-MWCNTs’ degradation. We analyzed biodegradation of two different types of ig-MWCNTs by human (THP-1) and murine (RAW264.7) macrophages. After 10 days of incubation, we studied nanoparticle localization within cells; isolated intra- and extracellular ig-MWCNTs were used for quantitative analysis. Ultrastructural and morphometric analysis were performed using transmission electron microscopy; electron diffraction was used for nanotube identification. To estimate chemical alterations, energy-dispersive X-ray spectroscopy and Raman spectroscopy were used. The study showed that both intra- and extracellular ig-MWCNTs undergo almost complete biodegradation, but in different ways: intracellular nanotubes become perforated and reduce to graphene flakes, while extracellular become thinner. We believe that the demonstrated variability in the destruction of ig-MWCNTs by cells suggests the possibility of creating nanomaterials with controlled biodegradation properties.

## 1. Introduction

Carbon nanotubes (CNTs) are nanoparticles with various applications and the prospect of further increases in their industrial production and use. Currently, 766 nanomaterials based on carbon nanotubes have been registered [1]. The majority of industrial carbon nanomaterials are based on ig-MWCNTs, which are used as catalysts, additives to polymers, bases for composite materials (by filling or coating them), components in electronic circuits, and even as plant growth stimulators in agriculture [2]. In addition to intentional production, MWCNTs can be formed accidentally during the combustion of automobile fuel [3], as well as naturally during forest fires [4]. Thus, there is a high probability of human contact with these nanoparticles through the respiratory system, skin or gastrointestinal tract if they somehow enter the environment. At the same time, available data indicate that MWCNTs do not belong to the category of harmless nanoparticles. Studies on rodents have shown that MWCNTs induce inflammation in the lungs and affect cells in a manner similar to asbestos, which is carcinogenic and cytotoxic [5]. Contact between MWCNTs and the gastrointestinal tract can lead to necrosis [6]. In 2015, carbon nanotubes were detected in bronchoalveolar lavage fluid and inside lung cells of asthmatic children [7]. CNTs were found in all samples and were similar to nanoparticles isolated from dust and automobile exhausts in large cities [8]. These findings strongly suggest that humans are regularly exposed to CNTs, and such contact may be hazardous.

All of this raises questions about the protective properties of living cells, their ability to eliminate MWCNTs, and the potential for humans to regulate this process. One of the possible ways of MWCNT elimination in the human organism is their destruction by professional phagocytes—neutrophils or macrophages. The main mechanism of MWCNT degradation by macrophages or neutrophils is oxidation by ROS (reactive oxygen species), which are produced by peroxidases: MPO (myeloperoxidase), NOX2 (NADPH oxidase 2), etc. [9,10]. It should be noted that in most of the above studies, phagocytosis of MWCNTs by cells and their degradation within the latter were shown. In addition, it is known that phagocytes are capable of producing reactive oxygen species extracellularly [11], but the degradation of non-internalized MWCNTs under the action of extracellular agents secreted by phagocytes has not been separately assessed.

It must be taken into account that due to the mild purification process and the lack of functionalization, mass produced ig-MWCNTs are highly heterogeneous in morphological characteristics, have numerous defects and impurities in their structure and are insoluble in water [12,13]. Naturally occurring and anthropogenic (such as those resulting from fuel combustion) unrefined MWCNTs are expected to have similar morphologies. Most studies on the biodegradation of l-MWCNTs have been conducted using nanoparticles synthesized specifically for scientific research; these are purified, standardized, and functionalized [14,15,16]. These l-MWCNTs often have a near-perfect chemical structure, with minimal structural defects and impurities. Such nanotubes are ideal for research because they make it easier to compare or track the effects they induce. However, they are unlikely to be found in large quantities in the environment and, therefore, are less likely to come into contact with living organisms. On the other hand, ig-MWCNTs are the most common nanomaterial that living organisms might encounter, yet the biodegradation of these nanotubes has hardly been studied. We hypothesized that the structural characteristics of ig-MWCNTs might activate distinct mechanisms and pathways for their destruction by phagocytes, different from those observed for l-MWCNTs. In previous research, we showed that ig-MWCNTs degrade when incubated with sodium hypochlorite (the primary protective agent of professional phagocytes), exhibiting degradation patterns not seen with l-MWCNTs: relatively uniform destruction of the entire outer and inner graphene layers was revealed [13]. This effect was accompanied by a decrease in defect density (instead of an increase, as was shown for l-MWCNTs), as demonstrated by Raman spectroscopy. We obtained a crucial finding that the wall thickness of ig-MWCNTs was not a determining factor for the degradation rate. Instead, the intensity of degradation depended on the nanomaterial’s initial properties, particularly the presence of impurities in the samples, which played a significant role in the process [13]. These experiments were conducted in an inanimate system, and targeted studies of ig-MWCNT degradation by neutrophils or macrophages had not been previously performed. In this context, the purpose of our study was to analyze the morphological and physicoсhemical changes in ig-MWCNTs under the influence of proinflammatory macrophages in vitro. Additionally, our study considered both extra- and intracellular localization of ig-MWCNTs. The nature of these alterations provides insight into the potential elimination mechanisms of ig-MWCNTs by macrophages, depending on whether they are intra- or extracellular. This approach allowed us to compare the results with those obtained earlier and to suggest common patterns of this process. We utilized both human and murine macrophages, along with two different types of ig-MWCNTs (representing two completely independent models), to uncover general patterns in their degradation. A detailed description of the experimental nanomaterials (referred to as MWCNT-T and MWCNT-D) has been provided previously [13].

In this context, ‘degradation’ refers to morphological changes in the nanotubes (con-firmed via morphometric analysis), as well as changes in their crystal structure, defect density, and oxygenation. Transmission analytical electron microscopy, Raman spectroscopy, and energy-dispersive X-ray spectroscopy were employed for this purpose.

It is important to note that the identification of carbon nanoparticles in biological samples faces a number of difficulties. Since biological samples consist mainly of carbon, elemental analysis does not allow one to reliably detect them. Second, cells may contain electron-dense inclusions that must be distinguished from nanoparticles. Also, the degradation and biotransformation products of MWCNTs themselves may visually differ from the original nanomaterial. In the present work, we used electron diffraction as a tool to identify MWCNTs.

## 2. Materials and Methods

### 2.1. Experimental Design

This study utilized two independent models of proinflammatory macrophages. Model 1 involved human macrophages (THP-1 cell line) and ‘Taunit’ ig-MWCNTs (MWCNT-T), while Model 2 involved murine macrophages (RAW264.7 cell line) and ‘Dealtom’ ig-MWCNTs (MWCNT-D).

**Figure 1:**
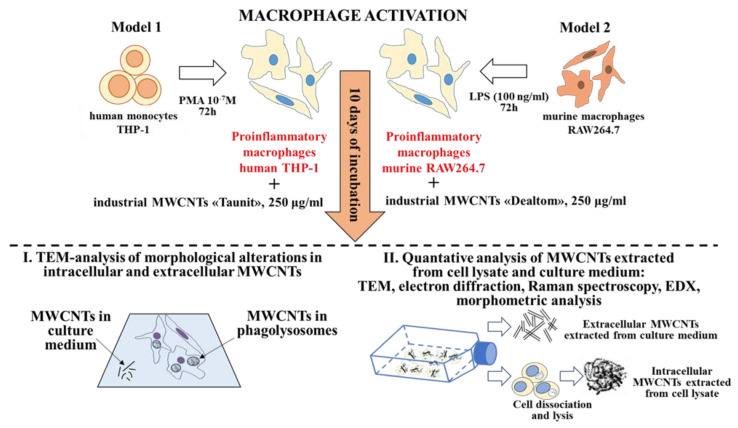
Experimental design and methods.

To identify morphometric and chemical changes in ig-MWCNTs, the following types of analysis were performed after incubating the experimental MWCNTs with macro-phages: (I) ultrastructural analysis of cells containing internalized MWCNTs; (II) analysis of MWCNTs isolated from cell lysates, as well as uninternalized MWCNTs isolated from the culture medium (Figure 1).

Additionally, we analyzed ig-MWCNTs treated with hydrogen peroxide in the presence of Fe^2+^ to simulate extracellular degradation.

### 2.2. Multi-Walled Carbon Nanotubes

**Figure 2:**
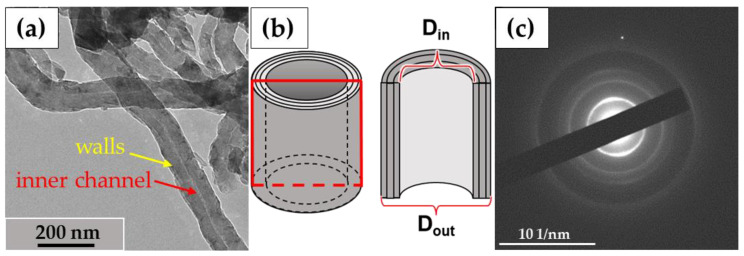
ig-MWCNTs: (**a**)—shape and form of nanotubes; (**b**)—scheme of the MWCNT’s structure. The red frame represents a longitudinal section of a single MWCNT; (**c**)—electron diffraction pattern for MWCNTs. Transmission electron microscopy.

In this study, we used the industrial carbon nanomaterials ‘Taunit’ (98.5% MWCNTs), produced by chemical vapor deposition (NanoTechCenter Ltd., Tambov, Russia), and ‘Dealtom’ (96.5% MWCNTs), produced by the high-pressure disproportionation technique (Centr Nanotecknologii, Moscow, Russia). Ni was used as a catalyst precursor for both nanomaterials. ((Taunit): https://zavkom.com/en/industries/other-industries/, accessed on 4 October 2024, (Dealtom): https://www.dealtom.ru/production, accessed on 4 October 2024). MWCNTs in their common form are a black, free-flowing fine powder. We have previously presented the technical characteristics of these nanomaterials [13]. Typical TEM images of the experimental MWCNTs and their electron diffraction patterns are shown in Figure 2.

### 2.3. Cell Lines

THP-1 is a monocyte-like cell line obtained from human peripheral blood with acute monocytic leukemia (Russian Cell Culture Collection, St. Petersburg Institute of Cytology, Russian Academy of Science). For THP-1 cultivation, we used RPMI-1640 medium supplemented with 2 mM L-glutamine and 10% fetal bovine serum (HyClone, Logan, UT, USA). Flasks containing the cell suspension were incubated in a CO_2_ incubator at 37 °C and 5% CO_2_. Actively proliferating cells required a partial medium change every 2–3 days.

RAW 264.7 is a macrophage-like cell line derived from a mouse tumor infected with the Abelson murine leukemia virus (the culture was provided by the Special Cell Technologies Laboratory, Moscow Institute of Physics and Technology). For RAW 264.7 cultivation, we used DMEM supplemented with 2 mM L-glutamine and 10% fetal bovine serum (HyClone, USA). Flasks containing the cell suspension were incubated in a CO_2_ incubator at 37 °C and 5% CO_2_.

### 2.4. Macrophage Differentiation

THP-1 cells were seeded in culture flasks (0.5 × 10^6^ cells/mL), and macrophage differentiation was induced by adding phorbol ester (Sigma, St. Louis, MO, USA) at an experimental concentration of 1 × 10^−7^ M, based on the data from Kurynina et al. (2018) [17]. At this concentration, PMA induces high phagocytic activity in the cells 72 h after differentiation induction. The culture medium was replaced 24 h after the addition of phorbol ester.

The RAW264.7 cell culture was differentiated into its pro-inflammatory phenotype by adding ultrapure lipopolysaccharides (LPS) from *E. coli* strain O111 (Invitrogen, Waltham, MA, USA) to the culture medium at a final concentration of 100 ng/mL. The addition of LPS has been shown to increase the phagocytic activity of RAW 264.7 cells [18]. The culture medium was replaced 24 h after LPS was added.

Cells were removed from the substrate and passaged using a cell scraper.

### 2.5. Isolation of MWCNTs from the Cell Culture Medium and Cell Lysate

MWCNTs (250 μg per 1 mL of culture medium) were added to the flasks 72 h after cell differentiation was induced. The cells were incubated with MWCNTs for 10 days, and the medium was partially changed on days 3 and 7 of incubation.

To collect unabsorbed extracellular MWCNTs after 10 days of incubation, the growth medium was taken, and the MWCNTs were isolated by repeated centrifugation (10,000 rpm, Eppendorf, Hamburg, Germany) and washed with distilled water (repeated 20 times).

To collect intracellular MWCNTs, the cells were detached from the substrate by adding trypsin solution. The resulting cell suspension was centrifuged at 1000 rpm for 30 min to sediment the cells. The precipitate was transferred into a microtube, and 500 μL of distilled water was added to the sediment. It was vortexed for 5 min to lyse the cells via osmotic shock. The cell lysate was further centrifuged (10,000 rpm), and the precipitate was heated in a thermostat at 85 °C for 30 min to remove cell debris, followed by several washes with distilled water and further centrifugation, as described by Elgrabli et al. [9].

### 2.6. Hydrogen Peroxide-Mediated MWCNT Degradation

MWCNT suspension (500 μg/mL) in distilled water was prepared in microtubes. Microaliquots of freshly prepared reagents were added once a day for 10 days. The reagent concentrations were 1 mM H_2_O_2_ and 20 μM Fe^2+^ (Merck, Darmstadt, Germany). After the last reagent addition, the MWCNTs were incubated for another 5 days. Incubation took place at room temperature on a shaker. After exposure, the MWCNTs were washed 30 times with distilled water through sequential resuspension and sedimentation in a centrifuge (10,000 rpm).

### 2.7. Transmission Electron Microscopy

After 10 days of incubation, cells were washed with 0.1 M PBS (pH 7.2–7.4) and fixed with 2.5% glutaraldehyde (Ted Pella, Inc., Redding, CA, USA) in 0.1 M PBS for 2 h. The cells were then washed to remove the fixative and subsequently fixed with a 1% solution of osmium tetroxide (OsO_4_) for 2 h. Samples were dehydrated in alcohols of ascending concentrations and acetone, then embedded in epoxy resin. Ultrathin sections (60–80 nm) were obtained using a LEICA UCT 4 ultramicrotome (Leica, Wetzlar, Germany) and stained with uranyl acetate and lead citrate, as described by Reynolds (1963) [19]. For the initial nanoparticle search and identification of MWCNT biodegradation products, individual samples were not subjected to Reynolds’ contrasting. The obtained sections were analyzed on a JEM 1400 microscope (JEOL, Tokyo, Japan).

### 2.8. Analytical TEM

For analytical TEM, nanoparticle suspensions (10 μL) were placed on a copper mesh covered with a formvar film and dried at room temperature.

#### 2.8.1. Electron Diffraction

Electron diffraction patterns provide crystallographic information about a material and determine different types of materials—amorphous, single- or polycrystalline. There are several types of electron diffraction patterns and their formation depends on the specimen (thickness, crystal structure, etc.). Ring patterns are created by polycrystalline materials. Interpretation of these patterns is used to determine crystalline phases in various materials. Using the radius of each ring, it is possible to specify the distance between the planes or interplanar spacing [20,21,22].

Although this method is used in crystallography and allows the obtaining of valuable information about the structure of nanoparticles, it can effectively be used for their detection in samples as well. Since the diffraction pattern (the number of rings and the distance between them) is specific to each nanomaterial, it is possible to obtain a reference electron diffraction pattern of the original nanomaterial and then compare it with the electron diffraction patterns of individual particles or their clusters, thus quickly identifying them [23].

Cell sections and isolated nanoparticles were examined using a ‘JEM 2100’ analytical electron microscope (JEOL, Japan) (200 kV, non-corrected LaB6 cathode). A Gatan FT1000 2k camera (Gatan, Pleasanton, CA, USA) was used.

To identify MWCNTs, the electron diffraction patterns of the nanoparticles were merged with a micrograph of a reference MWCNT electron diffraction pattern using Ado-be Photoshop CC 2019 (Adobe, San Jose, CA, USA). The criterion for MWCNT identification was the complete matching of the diffraction rings.

#### 2.8.2. EDS

EDS microanalysis was performed using a detector (Oxford Instruments Inca X-Max 8 mm^2^; Abingdon, Oxfordshire, UK) connected to the vacuum chamber of an analytical transmission electron microscope (JEM 2100, Tokyo, Japan) with Inca 4.15 software (Oxford Instruments, Abingdon, Oxfordshire, UK). The collected data were converted to Excel (Of-fice 2016, Microsoft, Redmond, WA, USA) from the original format. Each sample was measured in 15 areas, and Student’s *t*-test was used to compare independent samples.

### 2.9. Raman Spectroscopy

Raman spectroscopy was conducted using a confocal Raman microscope (inVia In-Spect, Renishaw, Wotton-under-Edge, UK). The recorded spectra covered the range from 0 to 3000 cm^–1^, using a diffraction grating with 1200 g/mm. Measurements were taken using a 50× objective and a 532 nm wavelength laser, with an intensity set to 1% of the maximum (0.7 mW). Samples were placed on aluminum foil, and 15 random points were analyzed for each sample; the spectrum acquisition time was 120 s per point. The heights of peaks D and G were then measured, and the I_D_/I_G_ ratio was calculated. Student’s *t*-test was used to compare independent samples.

### 2.10. Morphometric Analysis

To obtain morphometric data, numerous micrographs of both control and experimental MWCNTs were captured. Using the analytical tools of ImageProPlus 6.0.0.260 software (Media Cybernetics, Rockville, MD, USA), the outer and inner diameters were measured along the entire length of each nanotube (n ≥ 300 for each sample), and average values were calculated. Statistical calculations were carried out using the STATISTICA 10 program, and Student’s *t*-test was used to assess statistical significance.

## 3. Results

### 3.1. Ultrastructural Analysis of the Localization of ig-MWCNTs in Human and Murine Macrophages

#### 3.1.1. THP-1 Macrophages/ig-MWCNT-T

**Figure 3:**
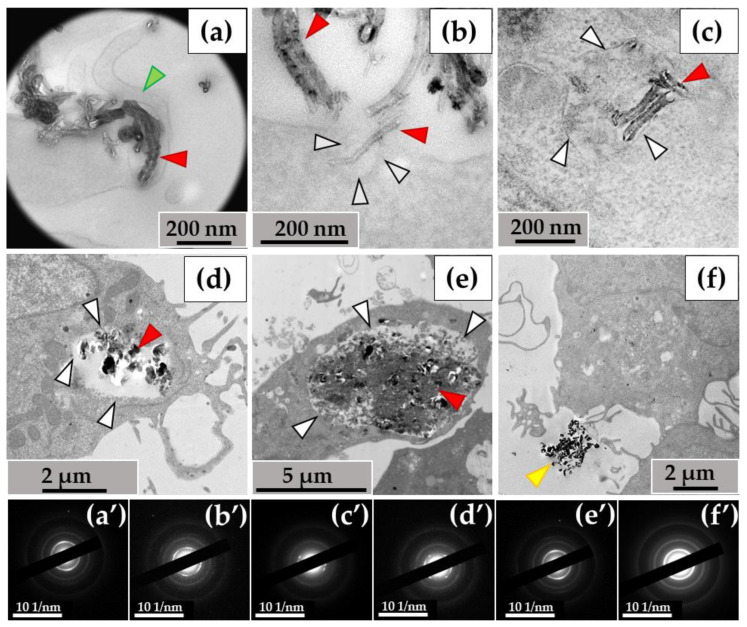
MWCNT-T localization in THP-1 macrophages, 10 days of incubation: (**a**)—phagocytosis of MWCNTs; (**b**)—MWCNTs enter the cell via membrane invaginations (arrow); (**c**–**e**)—MWCNTs in phagolysosomes; (**f**)—uninternalized MWCNTs. Transmission electron microscopy. (**a′**–**f′**) show electron diffraction patterns obtained from electron-dense clusters. Green arrows show cytoplasmic protrusions, white arrows phagosome membranes, yellow arrows non-internalized MWCNTs. MWCNTs are shown by red arrows.

When examining the ultrastructure of THP-1 macrophages, numerous pseudopodia were observed surrounding the nanotubes (Figure 3a) or invaginations of the cytoplasmic membrane where these nanoparticles were localized (Figure 3b). The cytoplasm of the cells contained numerous phagosomes/phagolysosomes filled with nanoparticles; their sizes varied significantly across individual ultrathin sections: small (0.2–0.8 μm) (Figure 3c), medium (1–2 µm) (Figure 3d), and large (5–10 µm) (Figure 3e). Clusters of MWCNTs were also located extracellularly (Figure 3f). Electron diffraction patterns were obtained from all electron-dense objects analyzed, and the crystal structure of the MWCNTs was confirmed (Figure 3a′–f′).

#### 3.1.2. RAW264.7 Macrophages/ig-MWCNT-D

**Figure 4:**
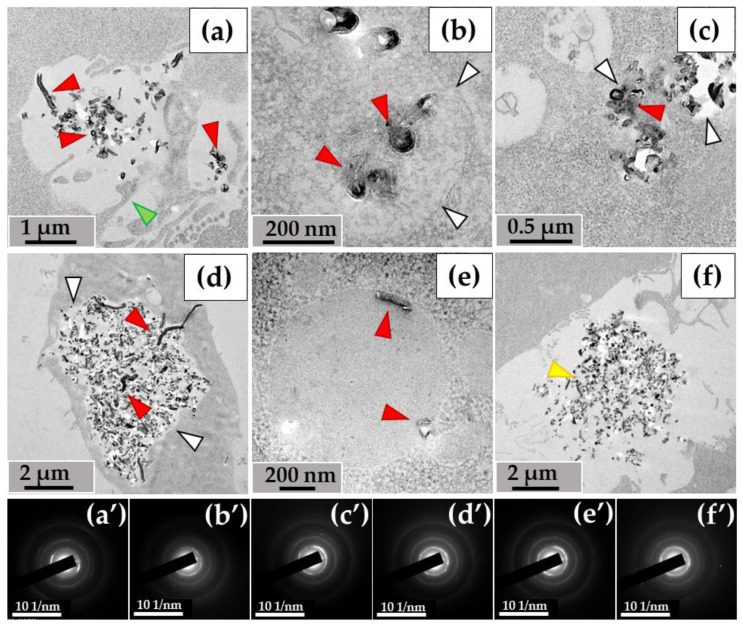
MWCNT-D localization in RAW264.7 macrophages, 10 days of incubation: (**a**)—phagocytosis of MWCNTs; (**b**–**d**)—MWCNTs in phagolysosomes; (**e**)—MWCNT in lipid droplet; (**f**)—uninternalized MWCNTs. Transmission electron microscopy. (**a′**–**f′**) show electron diffraction patterns obtained from electron-dense clusters. Green arrows show cytoplasmic protrusions, white arrows phagosome membranes, yellow arrows non-internalized MWCNTs. Internalized MWCNTs are shown by red arrows.

A similar pattern was observed with MWCNTs-D during incubation with RAW264.7 macrophages. Nanotubes entered the cells via phagocytosis (Figure 4a) and were detected in small (Figure 4b), medium (Figure 4c), and large (Figure 4d) phagosomes. Unlike MWCNT-T in THP-1 macrophages, MWCNT-D in RAW264.7 macrophages were also localized in lipid droplets (Figure 4e). Some nanotubes, like in the case of MWCNTs-T, were not subjected to phagocytosis and remained in close proximity to the cells (Figure 4f).

Thus, both experimental models were characterized by the entry of MWCNTs into cells through endocytosis and their subsequent localization in phagosomes. MWCNT-D was also detected in lipid droplets, which may indicate greater hydrophobicity compared to MWCNT-T. Individual clusters of nanoparticles that were not absorbed remained in the extracellular environment.

### 3.2. Morphological Analysis of Intra- and Extracellular ig-MWCNTs in Ultrathin Sections

#### 3.2.1. THP-1 Macrophages/ig-MWCNT-T

**Figure 5:**
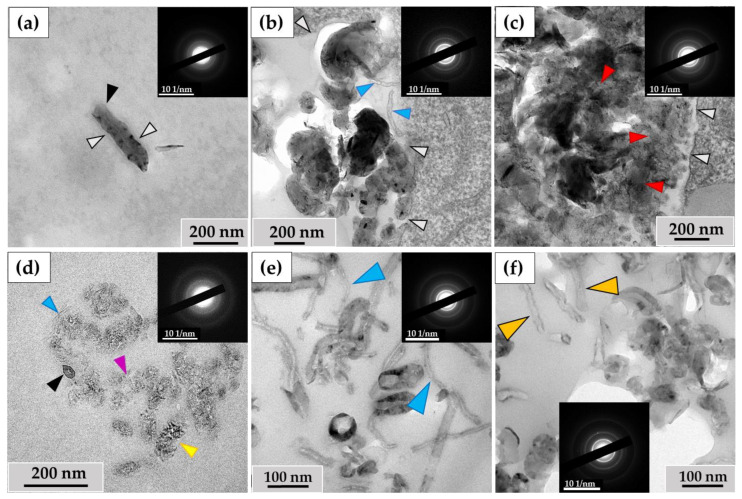
Morphology of MWCNTs-T after 10 days of incubation with THP-1 macrophages: (**a**)—MWCNTs in phagosome without significant alterations (black arrow); (**b**)—thinned MWCNTs (blue arrows) in phagosome; (**c**)—phagosome containing large cluster of MWCNTs with a poorly distinguishable structure of individual nanotubes (red arrows). White arrows indicate the boundaries of the phagosome; (**d**)—MWCNTs that retained their morphology (black arrow), thinned nanotube (blue arrow), perforated nanotube fragment (yellow arrow) and “graphene flakes” (purple arrow); (**e**)—fraction of thinned extracellular MWCNTs (blue arrows), (**f**)—extracellular MWCNTs with widened internal channel (orange arrows). The insets show electron diffraction patterns from the analysis area.

The ultrastructural investigation of MWCNTs in phagosomes revealed: (I) nanotubes without significant changes (Figure 5a); (II) clusters of electron-dense particles with morphologies different from intact MWCNTs, among which we identified (IIa) MWCNTs with thinned walls (Figure 5b, indicated by blue arrows) and (IIb) clusters of MWCNTs in which individual nanotubes were indistinguishable (Figure 5c); (III) fragments of MWCNTs with an elongated shape and non-uniform electron density, which we consider to be perforations in the walls, surrounded by electron-dense clusters of nano-particles with no recognizable MWCNT morphology. We refer to these particles as ‘graphene flakes’, as they are fragments of the graphene cylinders that form MWCNTs (Figure 5d). Notably, extracellularly localized MWCNTs also exhibited non-standard morphologies, including both very thin nanotubes (<20 nm) (Figure 5e) and nanotubes with widened internal channels (Figure 5f). All of these forms retained their crystal lattices, as demonstrated by electron diffraction patterns (Figure 5, insets).

#### 3.2.2. RAW264.7 Macrophages/ig-MWCNT-D

**Figure 6:**
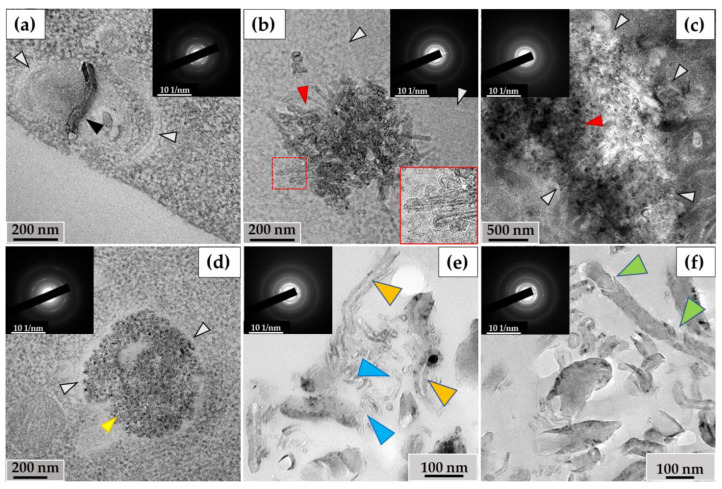
Morphology of MWCNT-D in RAW264.7 macrophages after 10 days of incubation: (**a**)—MWCNTs (black arrow) in phagosome without significant alterations; (**b**)—phagosome containing cluster of thinned nanotubes with distorted walls (red arrow); (**c**)—large cluster of MWCNTs with a poorly distinguishable structure of individual nanotubes (red arrow); (**d**)—“graphene flakes” (yellow arrow). White arrows (**a**–**d**) indicate the boundaries of the phagosome; (**e**)—extracellular fraction of both thinned MWCNTs (blue arrows) and MWCNTs with widened internal channel (orange arrows); (**f**)—extracellular MWCNT with damaged walls (green arrows). The insets show electron diffraction patterns from the analysis area.

In RAW264.7 cells, we observed MWCNTs with no significant alterations (mainly in small phagosomes) (Figure 6a) and clusters of highly thinned nanotubes (these MWCNTs had a thickness of <20 nm and heterogeneous electron density in their walls) (Figure 6b). In some large phagosomes, the structure of the MWCNTs was poorly distinguishable (Figure 6c). Similar to the THP-1/MWCNT-T model, clusters of nanoparticles without morphological signs of nanotubes, referred to as ‘graphene flakes’, were detected in RAW264.7 phagosomes (Figure 6d). These nanoparticles exhibited electron diffraction patterns that matched those of reference MWCNTs. Among the extracellular MWCNTs, both nanotubes with widened internal channels and ultrathin nanotubes were observed (Figure 6e). Additionally, some extracellular nanotubes exhibited localized breaches in their walls (Figure 6f).

Thus, the ultrastructural analysis of macrophages revealed qualitative changes in the structure of both intracellular and extracellular MWCNTs. However, performing a quantitative analysis of the observed morphological changes in MWCNTs on ultrathin sections of cells is challenging. For this reason, nanotubes were extracted from the cell lysate and growth medium for morphometric, structural, and elemental analyses.

### 3.3. Analysis of the Ultrastructure of ig-MWCNTs Isolated from Cell Lysate and Extracellular Medium

**Figure 7:**
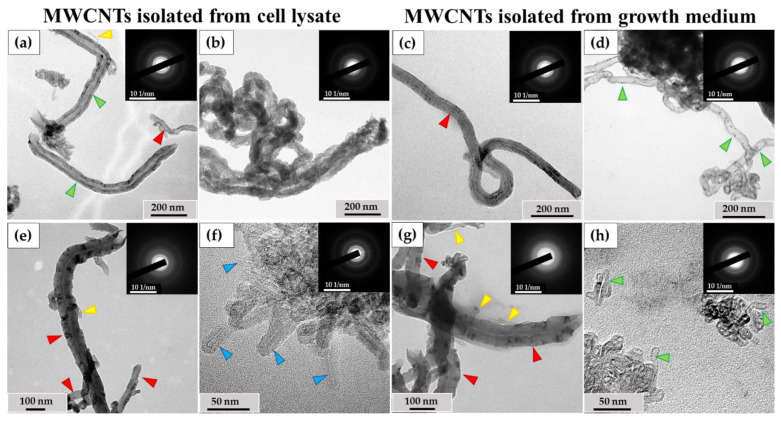
Isolated ig-MWCNT after incubation with macrophages. (**a**–**d**)—isolated MWCNT-T after incubation with THP-1 macrophages. (**a**,**b**)—obtained from cell lysate. (**a**)—MWCNTs-T without visible changes (red arrows), thinned nanotubes (yellow arrows) or nanotubes with locally widened internal channel (green arrows); (**b**)—MWCNTs-T with an altered structure, with numerous wall perforations and without visible internal channel. (**c**,**d**)—isolated from the growth medium. (**c**)—MWCNTs-T without visible changes (red arrows); (**d**)—MWCNTs with very thinned walls along the entire length and widened internal channel (green arrows). (**e**–**h**)—isolated MWCNTs-D after incubation with RAW264.7 macrophages. (**e**,**f**)—obtained from cell lysate. (**e**)—MWCNTs-D without visible changes (red arrows) and short thinned nanotubes (yellow arrows); (**f**)—cluster of MWCNTs-D with an altered structure—highly thinned with distorted wall structure (blue arrows); (**g**,**h**)—MWCNTs isolated from the growth medium: (**g**)—MWCNTs-D without visible changes (red arrows) or thinned (yellow arrows); (**h**)—short MWCNTs-D with thinned walls and widened internal channel (green arrows).

Among the MWCNTs-T isolated from the cell lysate, nanotubes were observed with no significant changes or with slightly widened internal channels (Figure 7a), along with severely deformed MWCNTs. These nanoparticles exhibited a sinuous shape, with no visible internal channels. They also displayed non-uniform electron density along their entire length, indicating the presence of numerous perforations in their walls (Figure 7b). The proportion of such nanotubes in the lysate was 9% of the total. These MWCNTs were frequently found in clusters and were morphologically similar to MWCNTs localized in phagolysosomes, as shown in Figure 5d. These nanoparticles also exhibited electron diffraction patterns that were consistent with MWCNTs, indicating that their crystal structure was preserved during morphological changes (insets).

An ultrastructural study was also conducted on unabsorbed MWCNTs isolated from the growth medium after incubation with macrophages. Among these MWCNTs, both nanotubes without visible changes (Figure 7c) and nanotubes with significantly thinned walls and widened internal channels (Figure 7d) were observed.

Among the MWCNTs-D isolated from the cell lysate, MWCNTs without significant alterations and very short, thinned MWCNTs (Figure 7e) were found. These thinned tubes were ≤10 nm in diameter and ≤100 nm in length and could only be observed at very high magnification. Additionally, clusters of thinned MWCNTs with damaged walls (Figure 7f) were detected. These nanotubes exhibited non-uniform electron density along their outer surface, indicating compromised graphene layer integrity, though the internal channel was preserved. The electron diffraction patterns confirmed the intact crystal structure of these MWCNTs. These nanotubes were only found in small clusters, and their proportion relative to normal morphology tubes could not be calculated.

Among the MWCNTs-D isolated from the extracellular medium, both nanotubes without visible changes in morphology and very thin nanotubes (similar to those in Figure 6e) were detected (Figure 7g). Clusters of short (≤50 nm) MWCNTs with widened internal channels and thinned walls were also identified (Figure 7h). However, the nature of these deformations differed from those observed in MWCNTs isolated from the cell lysate. The modified nanoparticles retained the MWCNT crystal structure.

### 3.4. Quantification of Changes in ig-MWCNTs after Incubation with Macrophages

To evaluate the quantitative changes in MWCNTs after incubation with macrophag-es, morphometric analysis, Raman spectroscopy, and energy-dispersive X-ray spectros-copy were used on MWCNTs obtained from: (I) control samples, (II) uninternalized MWCNTs, and (III) MWCNTs from cell lysates.

#### 3.4.1. Morphometric Analysis of ig-MWCNTs Isolated from Cell Lysate and Extracellular Medium

Excluding the fraction of perforated nanotubes, morphometric analysis of MWCNT-T isolated from the cell lysate did not reveal significant changes in either D_in_ or D_out_. In contrast, morphometric analysis of MWCNTs isolated from the culture medium showed significant wall thinning (up to 50%), from both the outer surface (decrease in D_out_) and the inner surface (increase in D_in_).

Significant changes in the outer diameter of nanotubes were observed in MWCNT-D, both in nanotubes obtained from the cell lysate and those from the extracellular medium. The degree of changes in the outer diameter was approximately the same in both intracellular and extracellular conditions (Table 1).

#### 3.4.2. Evaluation of Oxygen Content of ig-MWCNTs after Incubation with Macrophages

**Figure 8:**
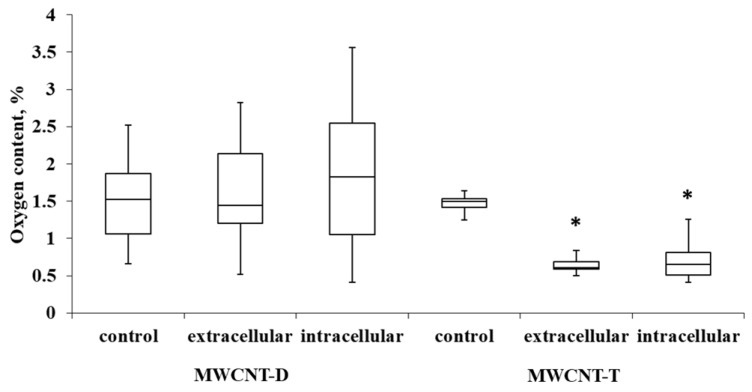
Oxygen content in samples of control MWCNTs, MWCNTs from cell lysate and uninternalized MWCNTs. Boxplots display the median and interquartile range (IQR; 25–75%) with whiskers representing the upper- and lower-quartile. Asterisks indicate statistically significant differences compared to the control sample (Student’s *t*-test).

For MWCNT-T incubated with THP-1, the initial oxygen content in control samples was 1.4%. It was observed that in the extracellular nanotube samples, the oxygen content was reduced by 2.04 times (0.7% oxygen in the sample) compared to the control. In intra-cellular MWCNTs, the oxygen content was reduced by 2.23 times (0.64% oxygen in the sample). MWCNT-D showed greater variability in oxygen content compared to MWCNT-T. After incubation, the variability increased, and no statistically significant differences could be established relative to the control (Figure 8).

#### 3.4.3. Evaluation of Defect Density of ig-MWCNTs after Incubation with Macrophages

Raman spectroscopy revealed a significant decrease in the I_D_/I_G_ intensity ratio by 1.15 times for MWCNT-T isolated from the cell lysate and by 1.22 times for unabsorbed MWCNTs.

**Figure 9:**
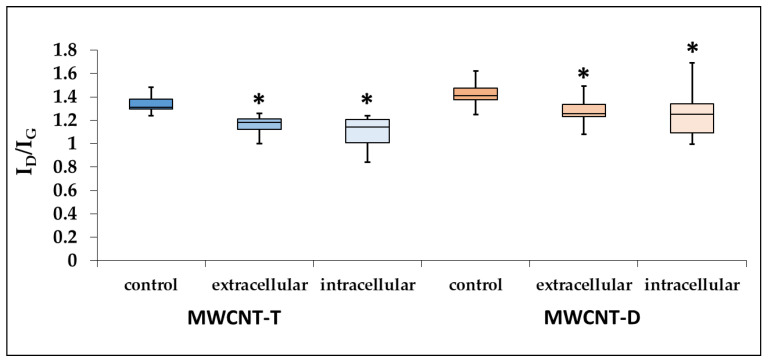
I_D_/I_G_ ratio for control MWCNTs, MWCNTs from cell lysate and uninternalized MWCNTs. Boxplots display the median and interquartile range (IQR; 25–75%) with whiskers representing the upper- and lower-quartile. Asterisk designates a statistically significant difference with control samples (Student’s *t*-test). Initial Raman spectra are given in Supplementary Materials (Figures S1–S6).

For RAW264.7, the defect density of MWCNT-D isolated from both the lysate and the culture medium also decreased compared to the control. Specifically, in the case of the cell lysate, the I_D_/I_G_ intensity ratio decreased significantly by 1.15 times, while in the case of unabsorbed MWCNTs, it decreased by 1.12 times (Figure 9).

To summarize this section (Section 3.4), it can be concluded that during the biodegradation of industrial MWCNTs by macrophages, a distinct tendency toward nanotube thinning and a decrease in defect density is observed. However, the data regarding changes in oxygen content are statistically significant only for ig-MWCNT-T.

### 3.5. Evaluation of Morphological Changes in ig-MWCNT Hydrogen Peroxide Treatment

In previous studies, we explored the effects of NaOCl and acidic pH on MWCNT degradation. Acidic conditions caused perforations in the nanotube walls, while sodium hypochlorite led to both external and internal wall thinning, widening the internal channel [13,24]. To further study ig-MWCNT degradation, we incubated our samples with hydrogen peroxide, a common cellular oxidizer used against pathogens.

**Figure 10:**
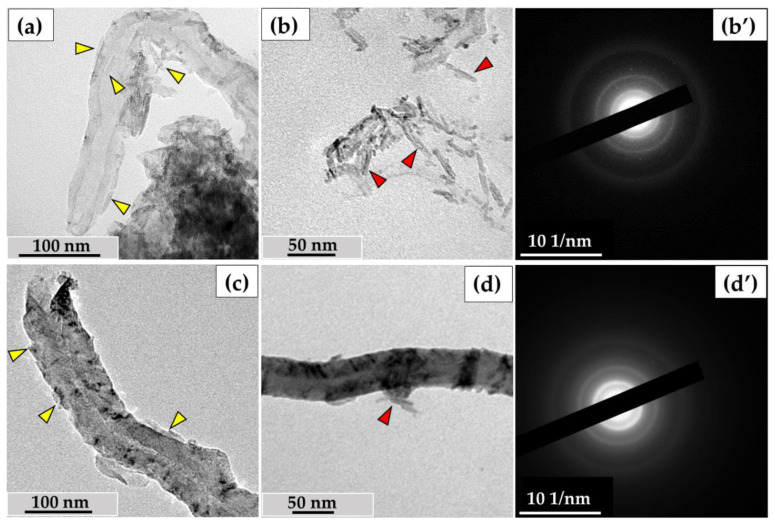
Degradation of ig-MWCNTs under the action of hydrogen peroxide in the presence of iron ions: (**a**–**b′**)—MWCNT-T, (**c**–**d′**)—MWCNT-D; (**a**,**c**)—scale-like graphene fragments peeling off the MWCNT’s walls after incubating with H_2_O_2_ with addition of Fe^2+^ (yellow arrows), (**b**,**d**)—ultrastructure of scale-like particles (red arrows), (**b′**,**d′**)—electron diffraction patterns.

After incubating MWCNTs-T with hydrogen peroxide in the presence of Fe²⁺, we observed scale-like particles, up to 50 nm long and 2-5 nm thick, around the nanotubes. These structures surrounded the nanotubes, adhering to their entire length internally and externally (Figure 10a), and also formed separate clusters up to 200 nm (Figure 10b). Elec-tron diffraction patterns of these particles remained consistent with those of MWCNTs (Figure 10b′), suggesting that each scale represents multiple layers of graphene exfoliated from the MWCNT surface.

A similar phenomenon was observed for MWCNTs-D—scale-like particles detached from the nanotube walls (Figure 10c) and adhered to the nanotubes (Figure 10d). Electron diffraction patterns of these particles did not show any additional diffraction rings or re-flexes beyond those of MWCNTs (Figure 10d′).

We measured the inner and outer diameters of the nanotubes after treatment with hydrogen peroxide to determine the intensity of wall thinning during the exfoliation of graphene flakes. However, the morphometric analysis did not show statistically significant differences in the wall thickness of either type of MWCNT after treatment compared to the control samples. This experiment suggests one possible mechanism for nanotube thinning, through the exfoliation of graphene flakes from either the outer or inner surface.

## 4. Discussion

From the results obtained, it follows that, unlike l-MWCNTs, when in contact with macrophages, heterogeneous ig-MWCNTs can undergo various types of degradation simultaneously, including thinning or perforation of the walls, decomposition into graphene fragments, or no degradation at all. This likely depends on the properties of individual nanotubes. It is important to note that the degradation of MWCNTs can be fully demonstrated only through a combination of research methods to confirm in each specific case whether the observed particle is a nanotube or a product of its biodegradation. Our findings also indicate that the degradation of ig-MWCNTs during incubation with living cells is only partially similar to what was previously observed in nonliving systems [13,24], suggesting that biological systems are more complex and may involve additional factors that influence nanotube degradation. Moreover, the direction and intensity of degradation depend on the extra- or intracellular localization of the nanotubes, as well as on the elimination strategies employed by macrophages. Some morphotypes of MWCNTs, such as very thin nanotubes, are found in both extracellular and intracellular fractions. We believe that some internalized MWCNTs may be released back into the growth medium following cell death, while MWCNTs that have undergone extracellular degradation may later be phagocytosed by cells.

Notably, even significantly transformed ig-MWCNTs and their biodegradation products retained electron diffraction patterns similar to those of control nanotubes. This observed effect suggests that until the very terminal stage of MWCNT degradation (to amorphous carbon or carbon dioxide), at least some part of the nanotube may retain its crystalline structure (several curved graphene layers), scattering electrons similarly to the original nanotube, while amorphous carbon loses its diffraction properties. This allows the identification of the products of MWCNT biodegradation, as well as detecting them among cellular organelles, until the very last stages. Additionally, since industrial MWCNTs may contain metallic impurities, the absence of additional diffraction rings also suggests that the observed nanoparticles are the result of nanotube degradation and not catalyst residues or contaminants.

As a result of our research, we found that both human and murine macrophages can reduce industrial multi-walled carbon nanotubes to graphene fragments upon contact with them. Using two independent models, we identified two general pathways for the effective elimination of MWCNTs during their exposure to active compounds produced by macrophages.

The first pathway occurs inside phagosomes/phagolysosomes, where thinned MWCNTs, MWCNTs with damaged graphene layers (perforations), and graphene ‘flakes’ are found within cells. The second pathway is typical for extracellularly localized MWCNTs, where MWCNTs lose graphene layers from the inner and/or outer sides, leading to wall thinning.

We propose that macrophages employ two different strategies to eliminate ig-MWCNTs, depending on their extra- or intracellular localization. This hypothesis was confirmed in two independent models of pro-inflammatory macrophages, utilizing two types of ig-MWCNTs.

One of the main functions of macrophages is to absorb, isolate, and destroy internalized objects when possible. To eliminate extracellular objects, macrophages can release oxidizing agents into the environment. Peroxidases and reactive oxygen species (ROS) play an essential role in the destruction of foreign objects, including nanotubes. Allen et al. (2008, 2009) [25,26], Russier et al., 2011 [27], and Zhao et al., 2011 [15] were the first to demonstrate the enzymatic oxidation and degradation of SWCNTs and l-MWCNTs using horseradish peroxidase and hydrogen peroxide (H_2_O_2_), showing that this process reduces nanotube length and degrades them to CO_2_ (as measured by gas chromatography) [26]. Further research revealed SWCNT destruction in vitro by mammalian peroxidases, such as myeloperoxidase (MPO) in neutrophils [10,28], eosinophil peroxidase (EPO) [29] and lactoperoxidase (LPO) [30] expressed by goblet cells. Most of this work was performed using isolated enzymes in nonliving systems, but these results indicate that various peroxidases can efficiently catalyze the degradation of any CNT within cells.

When macrophages absorb foreign objects, NADPH oxidase (NOX2), located on the plasma and phagolysosome membranes, becomes activated. NOX2 transfers electrons to oxygen, producing reactive oxygen species (ROS) such as hydrogen peroxide (H_2_O_2_), superoxide anion (O_2_^−^), and hydroxyl radical (OH^−^). During the respiratory burst, these ROS damage internalized objects, including MWCNTs [14,31]. Using liquid-phase transmission electron microscopy, Elgrabli et al. [14] observed ROS-mediated degradation of l-MWCNTs in situ and demonstrated that hydroxyl radicals, produced through water radiolysis, lead to two degradation mechanisms: a site-nonspecific process involving nanotube wall thinning and a site-specific process that transversely drills through existing defects. In the first mechanism, carbon–carbon double bonds are broken, forming carboxyl radicals; in the second, pre-existing defects react with oxidants. This process results in the formation of holes in the walls of l-MWCNTs.

Inhibition of MPO and ROS production leads to the absence of CNT biodegradation by macrophages [31,32]. Similarly, the severity of SWCNT degradation significantly decreased in lung macrophages of mice deficient in NADPH oxidase [33]. Thus, the available data indicate the decisive role of NADPH oxidase in the formation of ROS for the biodegradation of CNTs by macrophages. The supposed mechanism of CNT degradation is as follows: upon absorption of CNTs by macrophages, NOX2 produces O_2_·^−^. The latter is converted into H_2_O_2_ by superoxide dismutase (SOD) or reacts with free radical nitric oxide (NO·) to form peroxynitrite (ONOO^−^) [7,14]. Other peroxidases, such as MPO, catalyse the reaction of H_2_O_2_ with Cl^−^ to form hypochlorite [33]. In the presence of Fe^2+^, H_2_O_2_ converts to OH·. Peroxynitrite, hypochlorite, and hydroxyl radicals are capable of attacking CNT defects and unsaturated carbon bonds in the CNT walls [14], creating holes in graphene layers, which ultimately lead to the degradation of CNTs to CO_2_.

The important role of acidic pH in CNT degradation should also be noted. Vlasova et al. [34] showed an increase in the efficiency of hypochlorite-mediated SWCNT degradation at pH = 5.8 in comparison with pH = 7.4. Landry et al. [35] showed the dependence of the l-MWCNT degradation in macrophages on pH—after inhibition of the proton pumps (V-ATPase) by concanamycin, chemical changes in MWCNTs in macrophages were not detected by Raman spectroscopy.

Our data confirm the role of an acidic medium in this type of degradation. We have previously shown that murine gastric juice causes local changes in electron density in similar MWCNTs, which manifest as wall perforations and the disappearance of the inner channel [24]. These changes are similar to those we observed for ig-MCWNTs isolated from cell lysate. This indicates that even in the absence of specific ROS-generating enzymes, the acidic environment itself may lead to the emergence of defects in the structure of the MWCNT walls. We assume that highly defective ig-MCWNTs are targets for acidic treatment in phagolysosomes, where holes occur in areas of defects. At the same time, regions without initial defects appear to remain unaffected by such degradation. When analyzing MWCNTs from cell lysate (mainly from the phagolysosomes), we observed changes similar to the effect of gastric juice: the presence of perforated nanotubes with distorted graphene layers. We propose that this degradation depends on defect sites and occurs according to the following scheme: upon destruction of the defective section of the graphene cylinder, the next section of the cylinder becomes exposed to the active sub-stances in the phagolysosome. If there are no defects on the subsequent graphene layer, only a groove is formed in the MWCNT wall, and degradation in this region slows down. If a defect is present, a new hole is formed, thereby exposing the next graphene layer. At a high density of defects, the walls of MWCNTs can be destroyed to create through-holes. Furthermore, we assume that such tubes are reduced to “graphene flakes”.

We would like to draw attention to the existing contradiction regarding the activity of peroxidases in phagolysosomes. In most studies, the authors report an increase in the expression of NOX2 and MPO, suggesting a peroxidase-mediated mechanism for the intra-cellular degradation of MWCNTs. However, this contradicts data demonstrating that a decrease in pH deactivates these enzymes in phagolysosomes [36,37,38]. It should also be noted that NOX2 in antigen-presenting cells, including macrophages, can regulate pH in early phagosomes, thereby reducing the acidification of their internal environment. Thus, this issue has not yet been fully clarified.

According to existing data, the degradation of MWCNTs has been analyzed using the following methodological approaches: quantitative calculation of the nanotube amount in samples and assessment of defectiveness, as well as the presence of visible violations in the MWCNT structure. Among the main parameters characterizing the degradation of laboratory-grade MWCNTs, the following are distinguished: a decrease in length, an increase in the I_D_/I_G_ ratio, the appearance of additional oxygen-containing functional groups on the surface, wall thinning, and the formation of holes. In this context, the primary pathway for l-MWCNT degradation by cells is considered to be their internalization within phagolysosomes [9,35,39].

The data presented in our work differ from those reported above, as we used an industrial-grade carbon nanomaterial, which is highly heterogeneous in terms of the characteristics of the contained MWCNTs [13]. The main difference between our findings and the existing literature is a decrease in the overall level of defectiveness of ig-MWCNTs during their incubation with macrophages. This indicates that when the biologically active agents of macrophages damage these nanotubes, they specifically target sites of already existing structural defects and functionalization.

Notably, the vulnerability of MWCNTs to the effects of biologically active substances is largely determined by the presence of various defects in the crystal lattice and functional groups. Chemical reactivity, mechanical strength, and ability to transfer electrons all vary depending on the nature and quantity of these defects. Defects can represent both a compromised crystal structure of the nanotube and inclusions of other atoms. Despite the variety of defects in MWCNTs, all of them lead to disruptions in the chemical structure of the graphene layers and, consequently, a decrease in the strength of the entire crystal lattice or the emergence of individual stressed regions [40]. It is common to assume that such defective areas of industrial-grade nanotubes will be targets for ROS and other active compounds (acidic pH, ions etc.).

Based on the results obtained, we propose several stages of intracellular degradation of MWCNTs. The initial stage involves the internalization of MWCNTs by cells. As a result, MWCNTs are exposed to NOX2, MPO, and low pH in phagolysosomes. Under the influence of an aggressive acidic environment inside the phagolysosomes, the most defective nanotubes lose defect sites, leading to wall perforation; in this case, the I_D_/I_G_ ratio naturally decreases. Subsequently, the number of holes increases, ultimately resulting in the degradation of MWCNTs into graphene particles and, possibly, CO_2_. Due to the heterogeneity of the material, the least defective MWCNTs are more resistant to degradation, while the most defective ones undergo complete destruction. Similar results were observed by Nunes et al. (2012) when a suspension of functionalized MWCNTs was injected into the cerebral cortex. After two weeks of incubation, both unchanged MWCNTs and graphene-like fragments were detected in microglial cells. At the same time, similar to our findings, the I_D_/I_G_ ratio decreased, indicating changes in the chemical structure [41].

We have shown that during extracellular degradation, nanotubes are damaged uniformly—this effect may occur both from the inner channel and from the outer layers, which may indicate that this kind of oxidation is not site-specific. Similarly to intracellular degradation, the overall level of defectiveness significantly decreased in the samples. Obviously, the highest defect density may be located in the outermost and innermost graphene layers. A decrease in the amount of oxygen in MWCNT-T may indicate that most of the defects are related to oxygen-containing groups. These data are partially consistent with our earlier data on exposure to hypochlorite [13].

Despite the fact that the extra- and intracellular degradation of the two types of MWCNTs followed a similar pattern, differences were observed. During degradation in phagosomes, MWCNT-D retained their internal channel even in cases of wall structure distortion, while MWCNT-T nanotubes were completely perforated, resulting in the dis-appearance of the internal channel. During extracellular degradation, MWCNT-T lost both internal and external layers of graphene, while MWCNT-D became thinner only from the outside. Additionally, we did not observe significant differences in the change in oxygen content during the degradation of MWCNT-D. These effects can be explained by the fact that the nanotubes were synthesized under different conditions, according to the manufacturer’s data. This could lead to a situation where the defect sites for these nanotubes are chemically different from each other, and their distribution across the graphene layers is different.

Furthermore, we have shown that the effect of hydrogen peroxide with iron ions (Fenton reaction) leads to the peeling off of graphene layers from the inner and outer sur-faces of MWCNTs. According to González et al., when exposed to hydrogen peroxide with iron ions, the degradation of MWCNTs occurs as follows: first, defects accumulate in the outer graphene layer, after which this layer is peeled off the nanotube surface [16]. Figure 10 illustrates this situation; individual fragments of graphene cylinders peel off from the outer or inner surface in the form of scales. Sodium hypochlorite appears to have a similar effect, as nanotubes thin out relatively evenly, both outward and inward. However, we did not identify individual scale-like particles after hypochlorite treatment. Nevertheless, the oxidizing ability of hypochlorite is apparently much higher than that of hydrogen peroxide; in this case, exfoliated graphene fragments can be rapidly oxidized to CO_2_.

These data concerning extracellular nanotube degradation contradict findings obtained by Landry et al., who did not observe changes in MWCNTs by Raman spectroscopy when studying cell supernatants containing unabsorbed MWCNTs [35]. This difference can be attributed to either the duration of exposure or the difference in the initial defectiveness of the MWCNTs themselves.

Professional phagocytes, especially neutrophils, are known to create an extremely high local concentration of ROS in close proximity to the target [42]. Under such influence, active destruction of entire graphene layers occurs, leading to significant wall thinning of the nanotubes and a decrease in the overall defect density due to the elimination of oxygen-containing defect sites.

**Figure 11:**
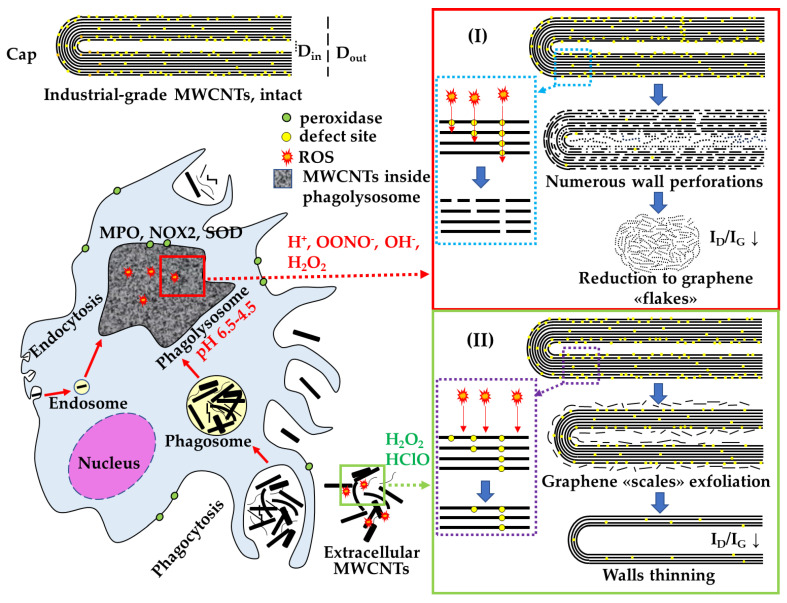
Scheme of extra- and intracellular effects of biologically active substances of macrophages on the MWCNTs degradation: I—site-specific degradation of MWCNTs in phagolysosomes; II—non-specific degradation of extracellular MWCNTs.

In summary, we have demonstrated that there are at least two pathways for the elimination of MWCNTs by macrophages, resulting in different morphological alterations. These pathways are intracellular, involving an acidic compartment, and extracellular, involving the release of ROS into the environment (Figure 11). The choice of a specific pathway is likely determined by the capability of cells to internalize MWCNTs.

At the same time, individual methods by themselves, be it TEM, analytical TEM or Raman spectroscopy, cannot give a complete picture of MWCNT degradation, and an objective assessment requires an integrated approach using all the above methods.

## 5. Conclusions

In this study, we demonstrated for the first time that, within two independent experimental models, macrophages are capable of degrading industrial-grade multi-walled carbon nanotubes in two parallel ways. We identified morphological changes in MWCNTs depending on their localization within or outside the cells. These morphological changes are associated with alterations in the internal channel, wall thickness, and integrity (the appearance of perforations), and are accompanied by changes in oxygen content and structural defect density (specifically, carbon atoms in sp^3^ hybridization). This allows us to propose that there are at least two mechanisms for the destruction of MWCNTs by macrophages: intracellular degradation, which is associated with the breakdown of MWCNTs into graphene flakes through wall perforation in phagolysosomes; and extracellular degradation, which involves the destruction of entire graphene layers and wall thinning.

Given the current trends towards increased nanoparticle production, it is likely that living systems, including humans, will encounter these materials more frequently. In this context, studying the mechanisms of carbon nanoparticle degradation is particularly relevant. The variability in the destruction of MWCNTs by cells observed in this study suggests the potential for creating nanomaterials with a controlled tendency toward biodegradation, which could enhance their biocompatibility while maintaining properties valuable for industrial use.

## Figures and Tables

**Table 1:** Morphometric analysis of ig-MWCNTs after 10 days of incubation with macrophages.

	RAW264.7/MWCNT-D			THP-1/MWCNT-T		
Diameter (D), nm	Control	Extracellular MWCNTs	Intracellular MWCNTs ^#^	Control	Extracellular MWCNTs	Intracellular MWCNTs ^#^
D_out_	69 ± 26	50 ± 28 *	47 ± 27 *	46 ± 15	35 ± 14 *	41 ± 17
D_in_	10 ± 3	8 ± 3	8 ± 3	9 ± 4	17 ± 7 *	9 ± 4
Average wall thickness	29 ± 12 (100%)	22 ± 14 (≈75%)	20 ± 11 (≈68%)	18 ± 7 (100%)	9 ± 5 * (≈50%)	16 ± 7 (≈89%)

^#^—MWCNTs with retained morphology. Data are shown as Mean ± SD. Statistically significant difference compared to control sample: *—*p* < 0.000001. Student’s *t*-test. n ≥ 300 for each sample.

## Data Availability

Data are contained within the article and Supplementary Materials.

## Supplementary Materials

The following supporting information can be downloaded at: https://www.mdpi.com/article/10.3390/nano14201616/s1, Raman spectra: Figures S1–S6.
