# Supplementary figures and images for: Biodegradation of Heterogeneous Industrial Multi-Walled Carbon Nanotubes by Pro-Inflammatory Macrophages
Source: Nanomaterials (Basel). 2024 Oct 10;14(20):1616. doi: 10.3390/nano14201616 (PMC11510322; doi:10.3390/nano14201616)

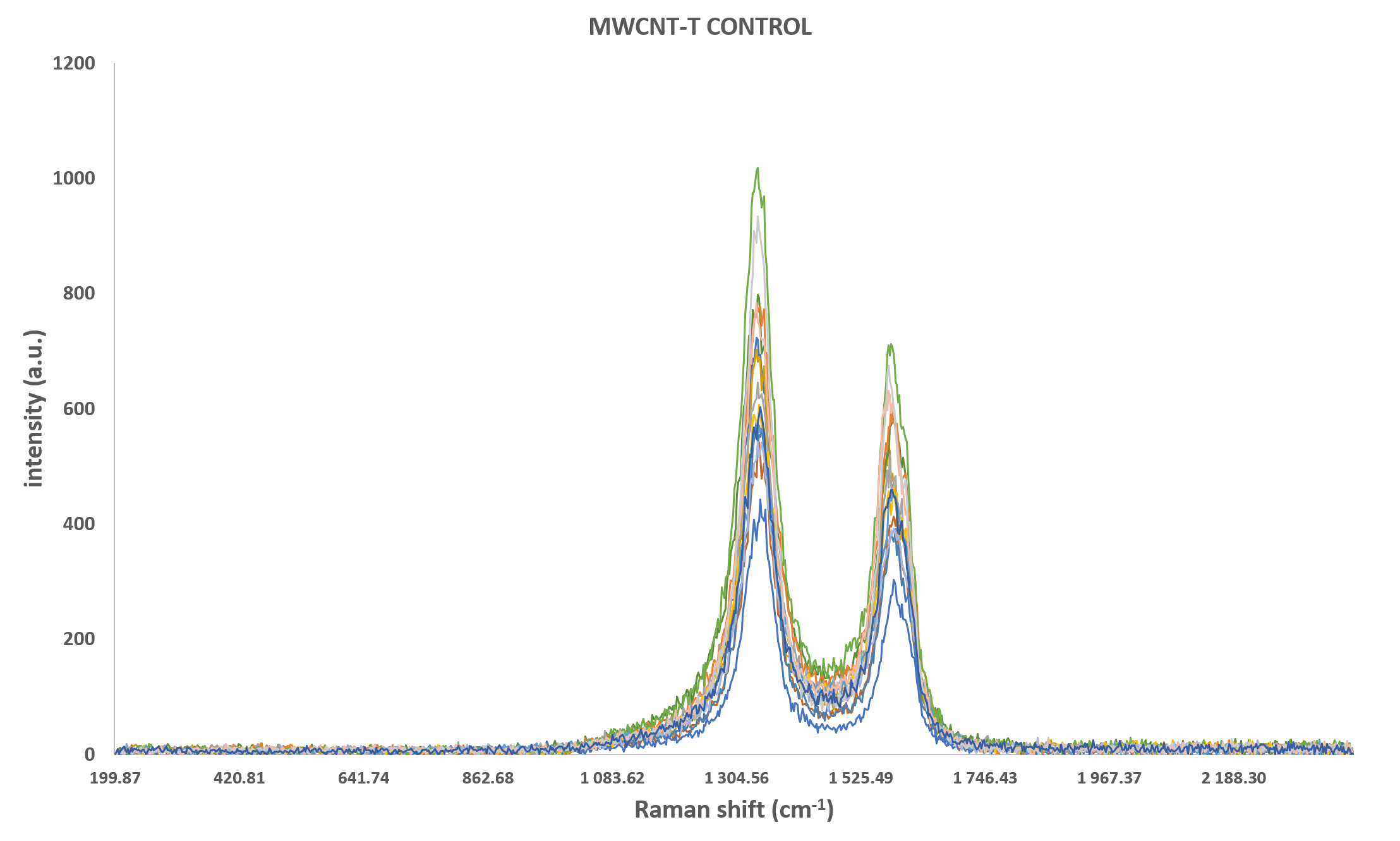

Supplement: Supplementary file 1 [file nanomaterials-14-01616-s001.zip › Figure S1.tif]

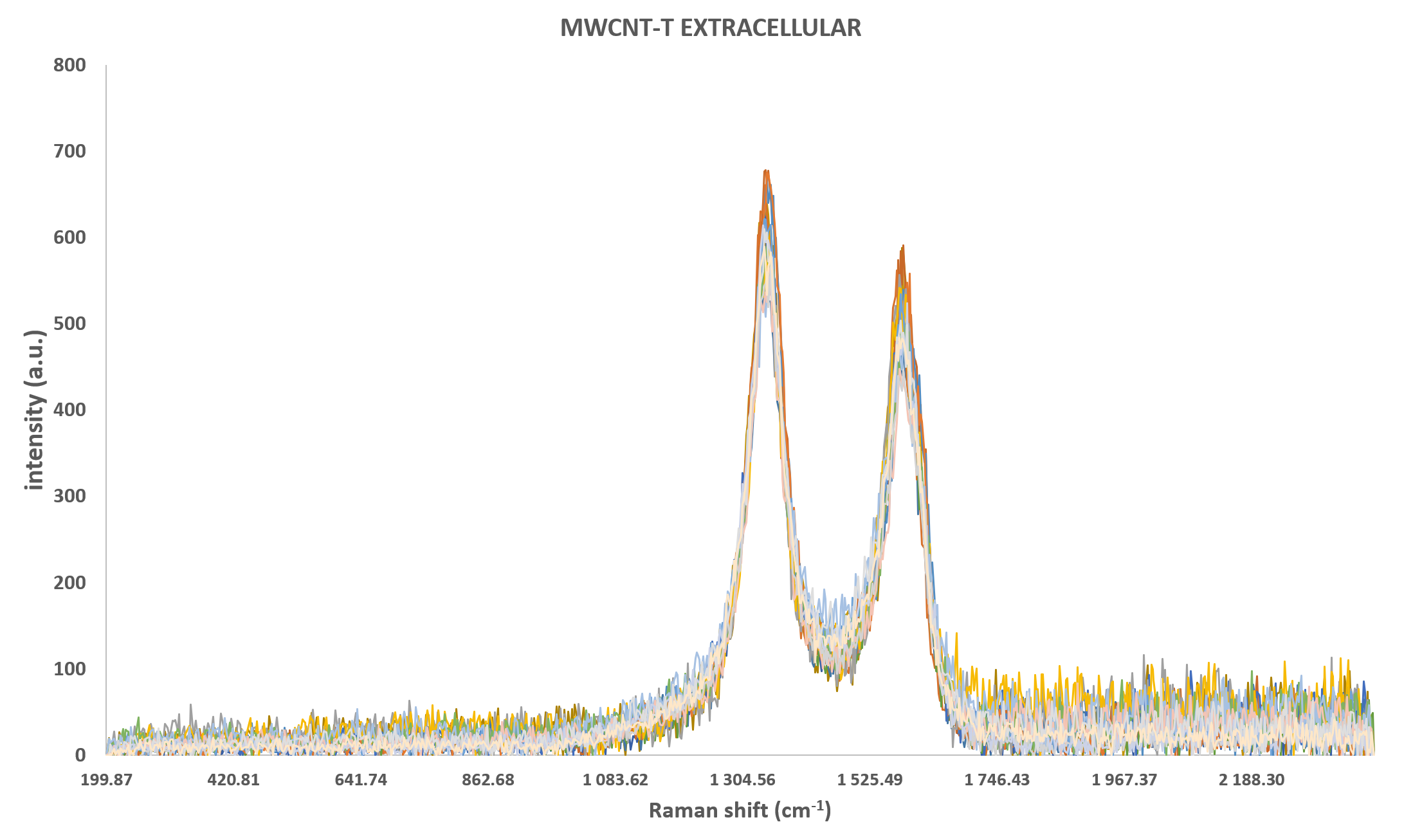

Supplement: Supplementary file 1 [file nanomaterials-14-01616-s001.zip › Figure S2.tif]

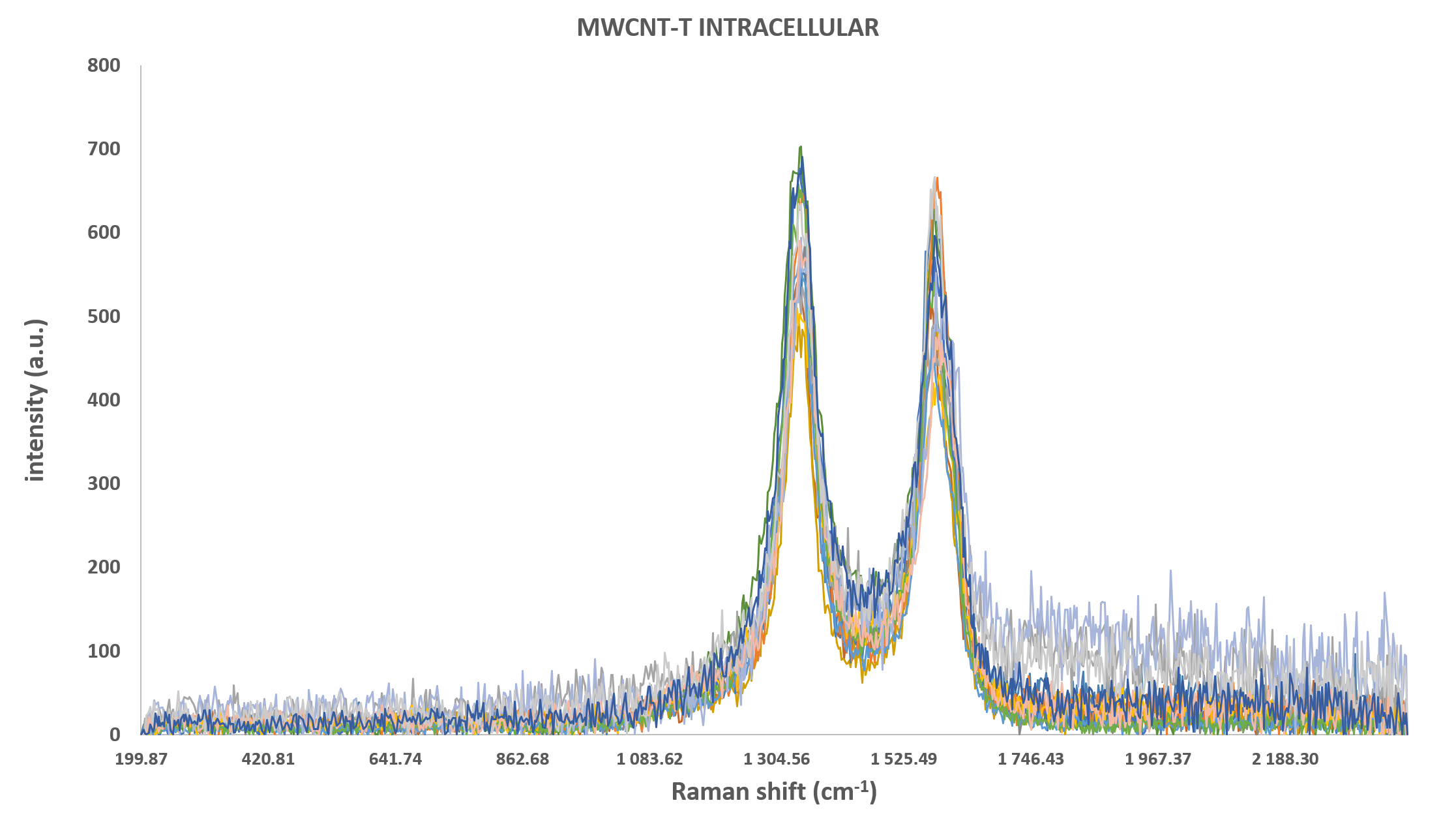

Supplement: Supplementary file 1 [file nanomaterials-14-01616-s001.zip › Figure S3.tif]

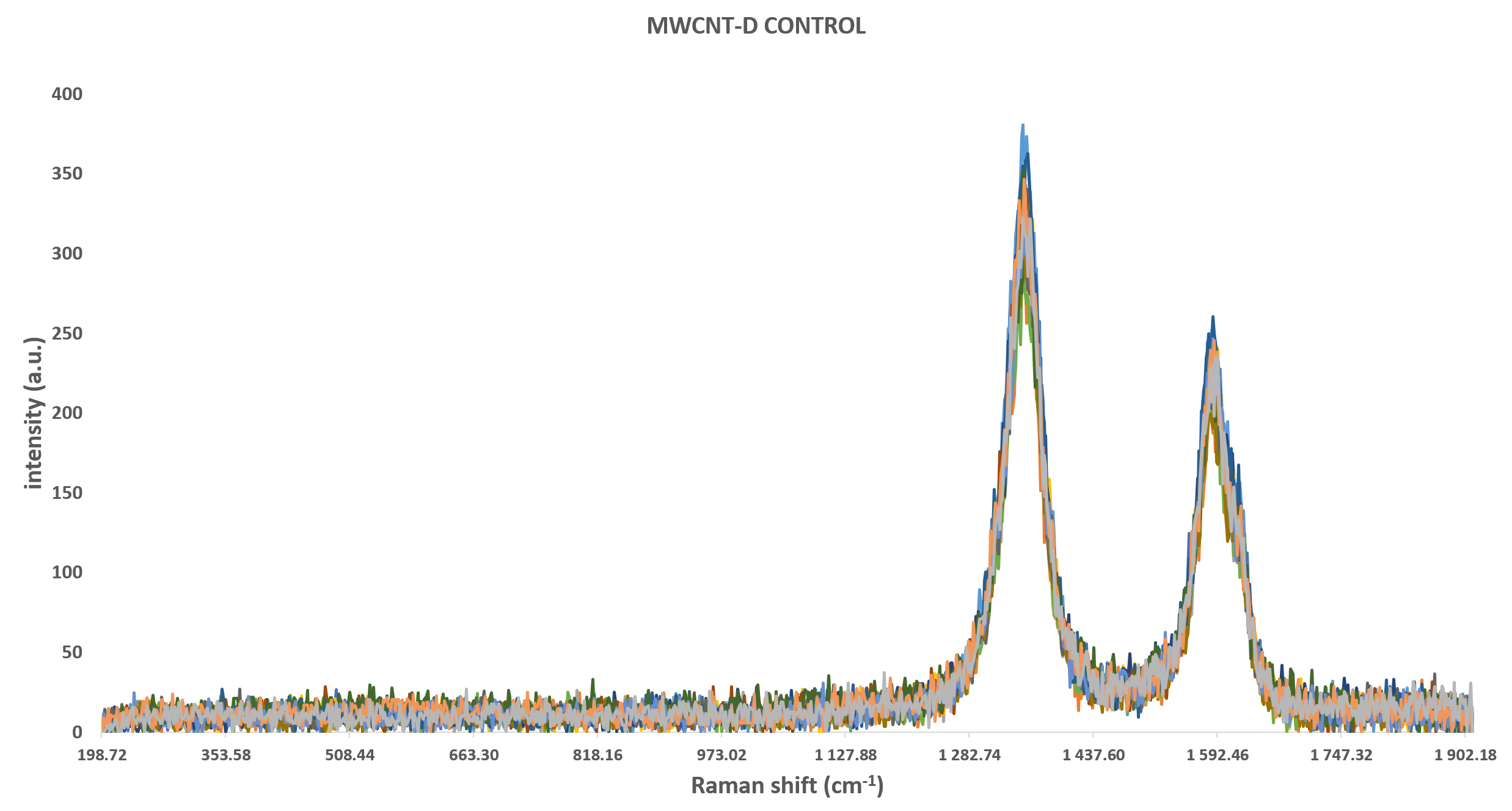

Supplement: Supplementary file 1 [file nanomaterials-14-01616-s001.zip › Figure S4.tif]

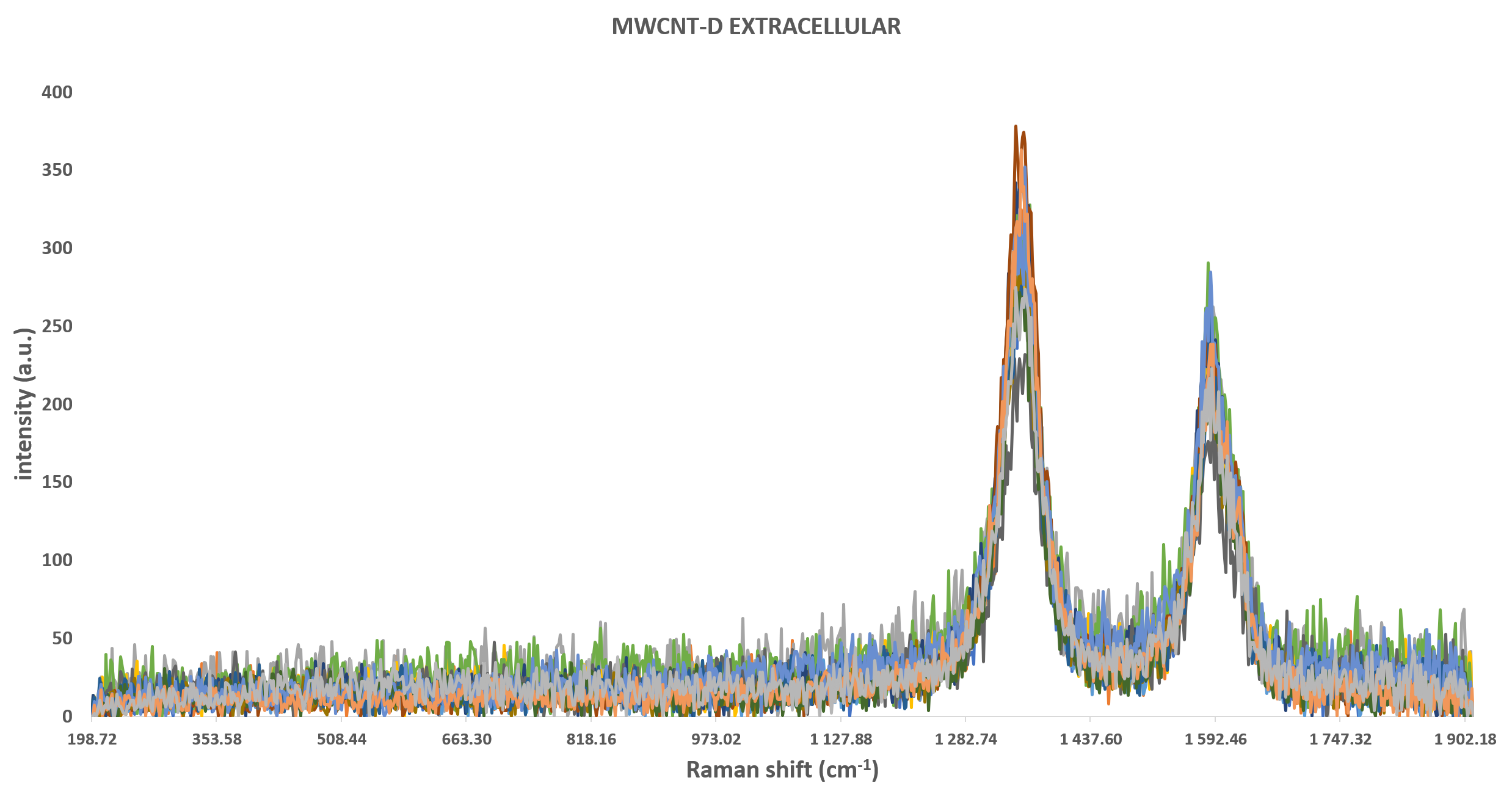

Supplement: Supplementary file 1 [file nanomaterials-14-01616-s001.zip › Figure S5.tif]

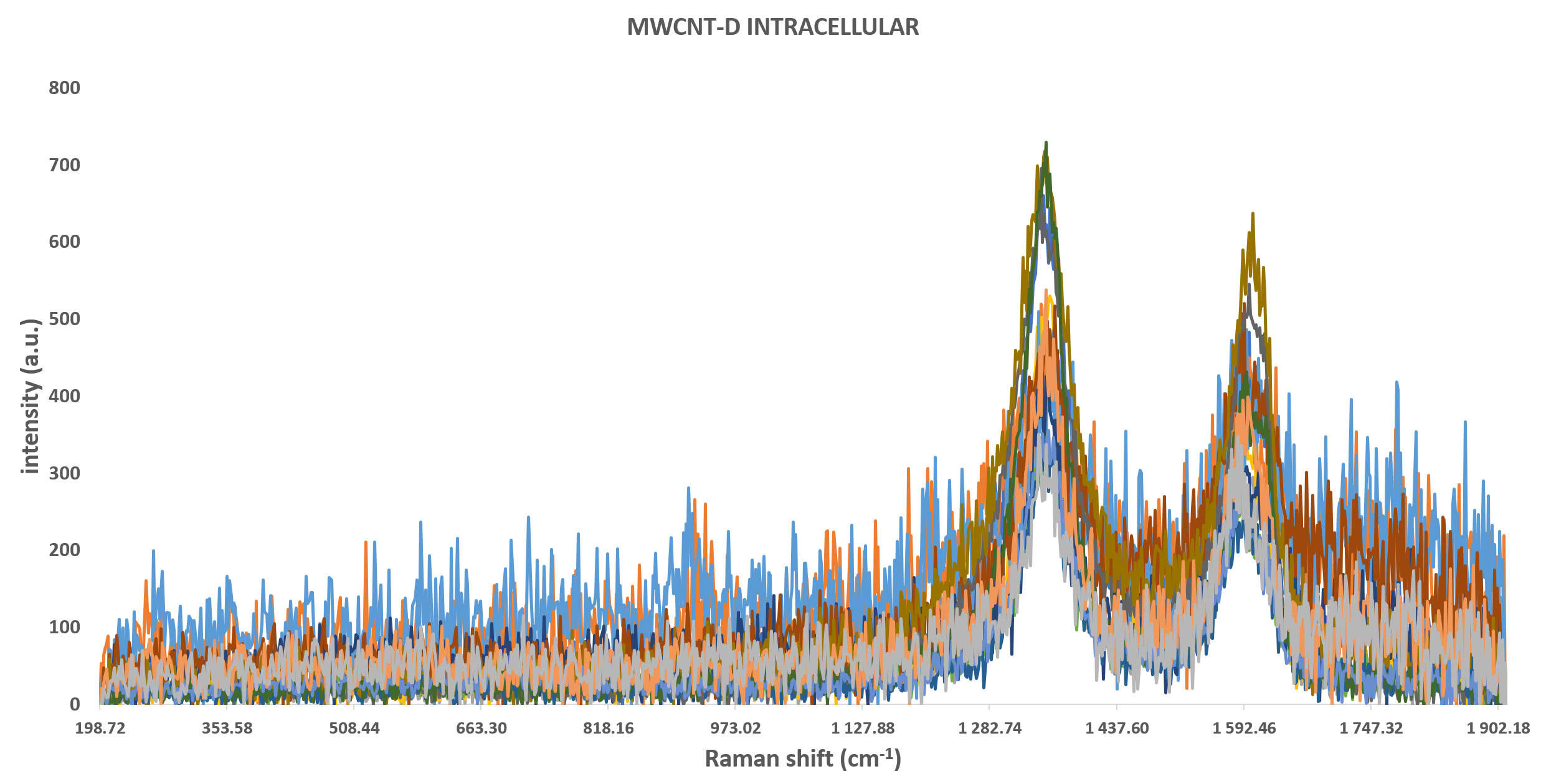

Supplement: Supplementary file 1 [file nanomaterials-14-01616-s001.zip › Figure S6.tif]
